# Critical Role of Mitochondrial Fatty Acid Metabolism in Normal Cell Function and Pathological Conditions

**DOI:** 10.3390/ijms25126498

**Published:** 2024-06-12

**Authors:** Sergey Dikalov, Alexander Panov, Anna Dikalova

**Affiliations:** Division of Clinical Pharmacology, Vanderbilt University Medical Center, 2220 Pierce Ave, PRB 554, Nashville, TN 37232, USA; alexander.panov55@gmail.com (A.P.); anna.dikalova@vanderbilt.edu (A.D.)

**Keywords:** fatty acid metabolism, mitochondria, respiration, pathological conditions

## Abstract

There is a “popular” belief that a fat-free diet is beneficial, supported by the scientific dogma indicating that high levels of fatty acids promote many pathological metabolic, cardiovascular, and neurodegenerative conditions. This dogma pressured scientists not to recognize the essential role of fatty acids in cellular metabolism and focus on the detrimental effects of fatty acids. In this work, we critically review several decades of studies and recent publications supporting the critical role of mitochondrial fatty acid metabolism in cellular homeostasis and many pathological conditions. Fatty acids are the primary fuel source and essential cell membrane building blocks from the origin of life. The essential cell membranes phospholipids were evolutionarily preserved from the earlier bacteria in human subjects. In the past century, the discovery of fatty acid metabolism was superseded by the epidemic growth of metabolic conditions and cardiovascular diseases. The association of fatty acids and pathological conditions is not due to their “harmful” effects but rather the result of impaired fatty acid metabolism and abnormal lifestyle. Mitochondrial dysfunction is linked to impaired metabolism and drives multiple pathological conditions. Despite metabolic flexibility, the loss of mitochondrial fatty acid oxidation cannot be fully compensated for by other sources of mitochondrial substrates, such as carbohydrates and amino acids, resulting in a pathogenic accumulation of long-chain fatty acids and a deficiency of medium-chain fatty acids. Despite popular belief, mitochondrial fatty acid oxidation is essential not only for energy-demanding organs such as the heart, skeletal muscle, and kidneys but also for metabolically “inactive” organs such as endothelial and epithelial cells. Recent studies indicate that the accumulation of long-chain fatty acids in specific organs and tissues support the impaired fatty acid oxidation in cell- and tissue-specific fashion. This work, therefore, provides a basis to challenge these established dogmas and articulate the need for a paradigm shift from the “pathogenic” role of fatty acids to the critical role of fatty acid oxidation. This is important to define the causative role of impaired mitochondrial fatty acid oxidation in specific pathological conditions and develop novel therapeutic approaches targeting mitochondrial fatty acid metabolism.

## 1. Introduction

In the past three decades, the rapid increase in metabolic conditions such as diabetes, lipid disorders, and metabolic syndrome has been paralleled by the epidemic growth of cardiovascular diseases [1,2]. This significantly increased the global burden of arterial and pulmonary hypertension, heart failure, stroke, and neurological diseases [3]. These pathological conditions are associated with the accumulation of fatty acids in circulation, intracellular deposits of lipid droplets, and excessive fat buildup in the body. Clinical studies support the causative role of fat accumulation in developing these pathological states [4]. Although these metabolic alterations have multiple origins, including environmental, genetic, and lifestyle origins, it has been suggested that lowering fatty acid consumption is beneficial, giving rise to the popularity of vegetarian and fat-free diets. This is further promoted by the studies indicating that a high-fat diet significantly increases cardiovascular diseases, feeding the scientific dogma that fatty acids drive many pathological conditions. As with many dogmas, this doctrine hinders further scientific research and development. In this work, we attempt to provide a balanced point of view in support of the fundamental role of fatty acid metabolism in normal cellular functions and a better understanding of how impaired fatty acid oxidation can promote pathological conditions.

Fatty acids were the primary energy source and essential cell membrane building blocks from the very origin of life. Earlier bacteria developed enzymatic machinery for highly efficient fatty acid oxidation to provide ATP and essential cellular metabolites. The evolutionary origin of eukaryotes was critically dependent on the development of specialized energy-producing organelle mitochondria. They were formed by the permanent enslavement of purple non-sulfur bacteria more than 1.45 billion years ago [5]. Mitochondrial evolution resulted in a highly integrated organelle comprising thousands of enzymes encoded in cellular nuclei. However, it has specific mitochondrial DNA, transmitted by only the maternal line. Although the main mitochondrial function is energy production, mitochondria are not just an “ATP-cow”. They have many functions: synthesis, cell signaling, cell growth, and cell death [6]. Mitochondria constantly cross-talk with the nuclei, critically affecting cell phenotype and function. Mitochondria can utilize various substrates, including amino acids, hydrocarbons, and fatty acids. Fatty acids represent the most energy-rich substrate, and their metabolism is evolutionarily preserved and critical for cellular functions of the organs constantly requiring large amounts of ATP. Despite recent progress in mitochondrial metabolism, we still lack an understanding of how mitochondria utilize several substrates simultaneously since most mechanistic in vitro studies were done in the presence of a single substrate. Long-chain and middle-chain fatty acids were almost excluded from in vitro studies. We know little about how mitochondrial metabolism is tuned for specific organs. We lack the in vivo mitochondrial functionality where the fatty acid oxidation occurs without oxidative stress observed in vitro.

One of the common confusions regarding fatty acids is the notion that our body does not require long-chain fatty acids. Indeed, vegetarian and fat-free diets can support most of our functions. Meanwhile, it is important to note that any consumption of proteins or carbohydrates leads to their metabolism in our body with the formation of fatty acids (except two essential fatty acids, alpha-linolenic acid and linoleic acid, that must be provided by food because these cannot be synthesized in the body, but they are critically necessary for health). Eukaryotic organisms can produce long-chain fatty acids from any available food/substrate. This provides great metabolic flexibility; however, we depend on fatty acid oxidation inside our body regardless of food, gender, and age. In all animals, whether herbivorous, carnivorous, or omnivorous, more than 90% of the energy required for the functions of the heart, kidneys, and skeletal muscles is provided by the β-oxidation of long-chain fatty acids.

**Figure 1:**
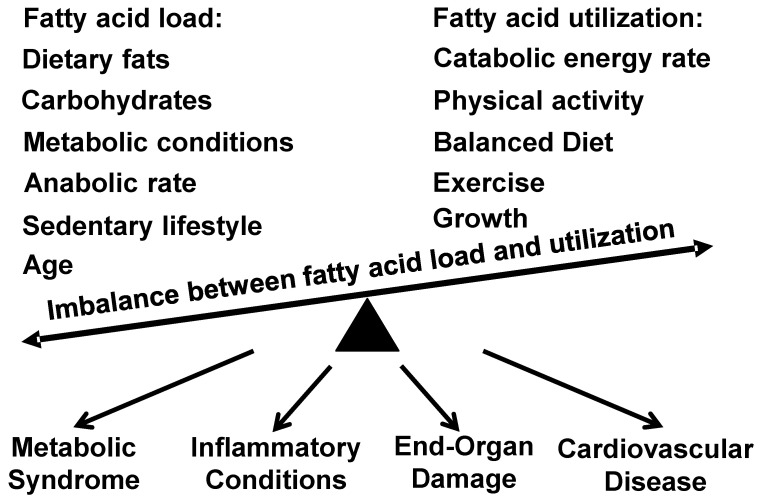
Pathogenic role of imbalance between fatty acid load and fatty acid utilization. Multiple genetic, physiological, environmental and lifestyle factors affect our body’s consumption and synthesis of fatty acids. Fatty acids are essential for basal catabolic energy production, body growth, and daily and physical activities. Multiple biological and social risk factors lead to imbalance between fatty acid uptake/production and utilization, resulting in harmful accumulation of long-chain fatty acids and promoting multiple pathological conditions.

The discussion above demonstrates that fatty acids are incorrectly accused of pathogenic effects since fatty acids are essential for our homeostasis. However, the imbalance between the fatty acid load and utilization can be implicated in pathological conditions. Several processes increase the fatty acid load, including intrinsic anabolic rate/synthetic activity of our body and risk factors such as metabolic conditions, sedentary lifestyle, and aging. Meanwhile, a high catabolic energy-burning rate can utilize all available fats. Other factors like tissue growth, physical activity, balanced diet, and exercise lead to a “lean” body due to extensive fatty acid utilization. The imbalance between the fatty acid load and the fatty acid utilization results in the harmful accumulation of long-chain fatty acids and promotes inflammation and tissue hypertrophy. It drives many pathological conditions (Figure 1). Understanding these specific processes on an individual basis can help to balance the fatty acid metabolism and reduce health risks and disease burden.

Several pathways are involved in the oxidation of different types of fatty acids in cells and the body. The gut microbiota partially transforms initial dietary lipids, breaking down dietary lipids to generate secondary metabolites with host modulatory properties [7]. For example, a large diversity of sphingolipids are synthesized by Bacteroidetes, some of which may affect the host’s physiology [7]. Recent studies report that gut microbiota is an important source of short-chain fatty acids, which play a significant role in regulating vascular functions. The therapeutic effect of short-chain fatty acids was reported in arterial and pulmonary hypertension [8,9]. Short-chain fatty acids, such as acetate, propionate, and butyrate, are made from the fermentation of dietary fiber and resistant starch in the gut. They modulate many metabolic pathways in the human body and affect obesity, insulin resistance, and type 2 diabetes [10]. Short-chain fatty acids can be increased by diet modulation and directly by supplementation, which can reduce cardiometabolic risks [11].

Long-chain fatty acids (20 to 14 carbon atoms) are the most abundant and supplied from food. Fatty acid β-oxidation shortens the acyl group and produces medium-chain fatty acids (12 to 8 carbon atoms). In contrast to long-chain fatty acids, diet is not the main source of medium-chain fatty acids, and they are mainly produced locally in cells and tissue [12]. Short-chain fatty acids comprise one to six carbons, mainly produced by gut microbiota [13]. It is important to note that all three classes of fatty acids are indispensable for human health. However, each type of fatty acid plays a very distinct role. Long-chain fatty acids are the main sources of energy and cellular building blocks. Medium-chain fatty acids are used both in energy production and metabolic synthetic pathways. Short-chain fatty acids are a minor source of energy (~10%) but have a tremendous regulatory role [14]. Therefore, seamless work of the highly orchestrated fatty acid oxidation is critical for balanced fatty acid metabolism, which also obligatorily requires the simultaneous presence of other mitochondrial metabolites such as amino acids and Kreb’s cycle intermediates [15].

Endoplasmic reticulum omega-oxidation of fatty acids processes large, water-insoluble fatty acids, which would otherwise be toxic to the cell in higher concentrations [16]. Peroxisomal α- and β-oxidation of fatty acids is involved in biosynthesis pathways [17]. Meanwhile, mitochondria fatty acid β-oxidation is the main cellular energy source critical for the normal function of most cells and tissues in our body. The sections below briefly review cell- and tissue-specific roles of mitochondrial fatty acid oxidation by addressing three critical questions. A. What is the role of fatty acid oxidation in normal function? B. What is the role of impaired fatty acid oxidation in pathological conditions? C. What is the therapeutic effect of targeting the fatty acid oxidation?

## 2. Heart

### 2.1. Fatty Acid Oxidation Supporting Normal Cardiac Function

**Figure 2:**
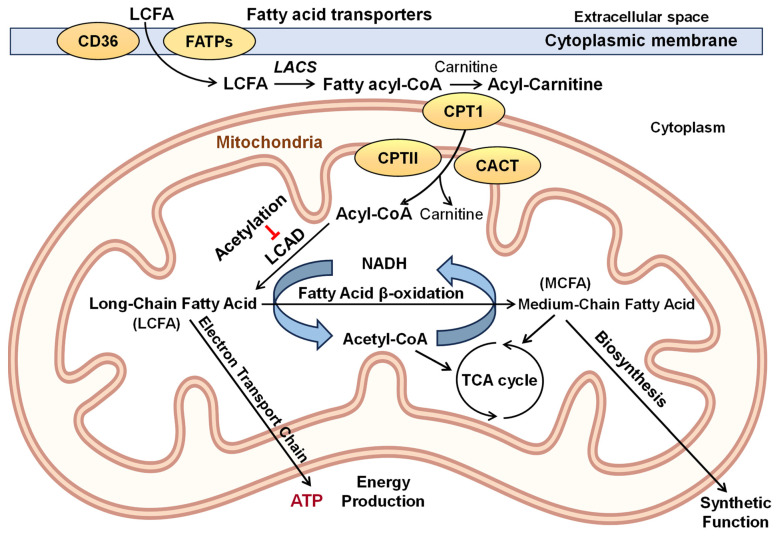
Schematic presentation of cellular fatty acid uptake and mitochondrial metabolism. Long-chain fatty acids (LCFA) enter the cells from circulation via cytoplasmic fatty acid transporters CD36 and FATPs. In cytoplasm, long-acyl CoA synthase (LACS) activates fatty acids to fatty acyl-CoA, which is combined with carnitine to produce acyl-carnitine for transport into the mitochondrial matrix by carnitine palmitoyltransferase I (CPT1) and carnitine-acylcarnitine translocase (CACT) where carnitine palmitoyltransferase II (CPT2) catalyzes the reconjugation of long-long-chain acylcarnitines to coenzyme A. In the mitochondrial matrix, long-chain acyl-CoA reacts with long-chain acyl dehydrogenase (LCAD), which initiates the fatty acid β-oxidation cycle. This produces acetyl-CoA, NADH, and medium-chain (C8-C12) fatty acids, driving energy production and the synthetic function of mitochondria. Metabolic conditions result in acetyl-CoA overproduction, leading to the acetylation of mitochondrial proteins such as LCAD, which inhibits fatty acid β-oxidation. Abbreviations: CACT—Carnitine-acylcarnitine translocase; LCAD—Long-chain fatty acyl-CoA dehydrogenase; TCA—Tricarboxylic acid cycle; NADH—Nicotinamide adenine dinucleotide reduced.

**Figure 3:**
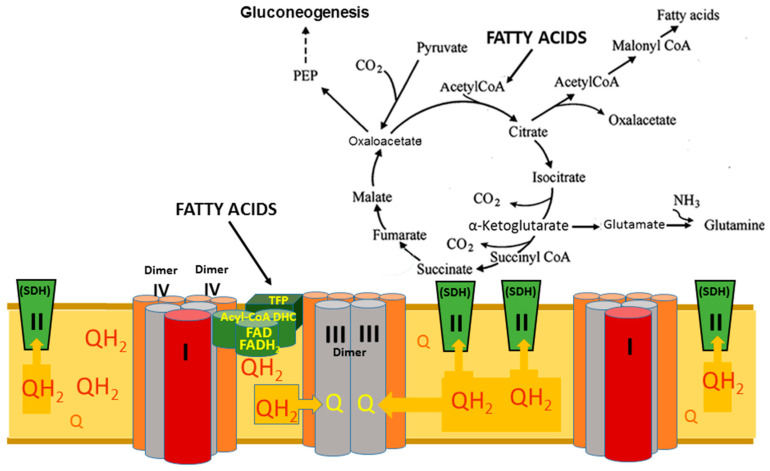
Functioning of respirasome and the Krebs Cycle during active β-oxidation of long-chain fatty acids. Abbreviations: Acyl-CoA DHC—acyl-CoA dehydrogenase complex, which includes three enzymes: acyl-CoA dehydrogenase, electron transfer flavoprotein (ETF), electron-transferring flavoprotein dehydrogenase (ETFDH); PEP—phosphoenolpyruvate; TFP—trifunctional protein of the oxidation of fatty acids system; SDH—succinate dehydrogenase; Q—ubiquinone, oxidized form of coenzyme Q; QH_2_—ubiquinol, reduced form of coenzyme Q. The Figure was adapted from Ref. [15].

Fatty acid oxidation accounts for 60–70% of oxygen consumption for energy production in the human heart [18]. This is mainly mediated by mitochondrial fatty acid β-oxidation. Fatty acid utilization is a very complex process. It includes cellular fatty acid uptake, cytoplasmic activation of fatty acids to acyl-carnitine derivatives, transport into the mitochondrial matrix by carnitine-palmitoyl transporter (CPT), and acyl-CoA formation [19]. These undergo four sequential reactions with long-chain acyl-CoA dehydrogenase, enoyl-CoA hydratase, 3-hydroxyl-acyl-CoA dehydrogenase, and 3-ketoacyl-CoA thiolase, resulting in the shortening of the fatty acyl-CoA by respiration and synthetic cellular pathways (Figure 2) [20]. The activity of fatty acid oxidation depends on the acetylation of these enzymes, such as long-chain acyl-CoA dehydrogenase (LCAD) [21]. Recent studies have shown that the enzymes of fatty acid β-oxidation are physically associated with the mitochondrial respirasome and organized into the complex functional superstructure [15]. Functional supercomplexes of the oxidative phosphorylation comprising respirasome, ATP-synthase, and other multienzyme complexes of associated metabolic pathways are held together in the negative bends of the inner leaf of the inner mitochondrial membrane with the help of cardiolipin and phosphatidylethanolamine (Figure 3) [15]. This makes the whole superstructure vulnerable to oxidative damage of these phospholipids by the protonated superoxide radical, namely perhydroxyl radical (HO_2_^•^) [22]. Thus, it is conceivable that dyslipidemia and other β-fatty acid oxidation disorders are preceded by hypoxia and other conditions that promote oxidative stress.

Roberts and Morrow from Vanderbilt University discovered the new nonenzymatic pathway of PUFA lipid peroxidation, named the isoprostane pathway of lipid peroxidation, which results in the formation of prostaglandin-like compounds with enormous variations in molecular positional and stereo-isomerism in structure and biological activities. A significant number of products of PUFA autoxidation initiated by perhydroxyl radical have very high reactivity with lipids and proteins, and the resulting products can be determined and initiated in the body’s tissues and fluids as one of the most reliable and sensitive early markers of oxidative damage of lipids and proteins. Cardiolipin and phosphatidylethanolamine are among the earliest targets for isoprostane lipid peroxidation [23,24,25]. According to Barja, water-soluble antioxidants are ineffective in preventing lipid peroxidation because the antioxidants work in the water phase, whereas the damages occur in the lipid phase [26]. The heart’s key metabolic feature is providing constant high energy to sustain its continuous contractile activity regardless of substrate availability [27]. This results in metabolic plasticity. The heart primarily oxidizes long-chain fatty acids but shifts to carbohydrates, lactate, pyruvate, and ketone bodies based on the metabolic environment and functional activity. For example, carbohydrate-rich meals increase blood glucose, and insulin favors glucose uptake and decreases fatty acid uptake by the heart. During fasting, fatty acids are preferential substrates due to their high concentration in the blood, increasing fatty acid contribution to energy production by up to 80%. This parallels a decrease in the utilization of carbohydrates. The contribution of fatty acids can be even more increased by fat-rich food intake. In case of limited oxygen supply, the glycolysis is increased, and the fatty acid oxidation is reduced to decrease oxygen consumption and minimize hypoxic injury [18]. This metabolic plasticity is impaired in pathological conditions, which will be discussed below.

### 2.2. Impaired Fatty Acid Oxidation in Cardiac Dysfunction

The described cellular fatty acid oxidation can be disrupted in multiple steps, and clinical studies support the causative role of impaired fatty acid metabolism in cardiac dysfunction. Alterations of diet, lifestyle, genetic mutations, metabolic conditions, and aging can lead to either accumulation of fatty acids or inadequate fatty acid oxidation, promoting cardiac dysfunction. In this section, we will review these pathogenic pathways.

Several human studies indicate that a diet high in saturated fats, trans fat, and cholesterol has been linked to heart disease. For example, geographic differences in mortality from coronary heart disease and stroke were associated with local dietary preferences within Europe [28]. Diets high in saturated fatty acids and trans fatty acids increase LDL cholesterol levels and, in turn, the risk of heart disease potentially due to impaired mitochondrial function and increased cardiovascular inflammation [29]. A high-fat diet increases the risk of heart failure and arrhythmias due to cardiac lipid overload [30]. Because normal β-oxidation of fatty acids requires the presence of supporting substrates [15], cardiac metabolism requires a balanced diet of fat, amino acids, and carbohydrates. The deleterious effects of an unbalanced “Western” diet have been extensively documented [31,32]. There is no “one-size-fits-all”, and appropriate fat consumption is highly variable. It depends on genetics, ethnic background, region, physical activity, and gender [33].

Genetic defects in fatty acid oxidation enzymes promote heart failure, cardiomyopathy, and sudden death [34,35]. This can be mediated by inadequate cellular fatty acid uptake by the CD36 transporter, defects in acyl-carnitine transport of fatty acid derivatives into mitochondria, and deficiency of fatty β-oxidation enzymes, leading to impaired energy metabolism and severe energy deficiency [36]. Meanwhile, genetic defects in fatty acid oxidation are responsible for only a fraction of cardiac disease. However, many known lifestyle and metabolic risk factors “mimic” genetic alterations and are responsible for most cardiac conditions. For example, smoking and a sedentary lifestyle impair fatty acid oxidation and promote cardiovascular disease [37,38]. Metabolic conditions such as diabetes and metabolic syndrome are linked to mitochondrial fatty acid β-oxidation alterations and increase heart failure, ischemic heart disease, and diabetic cardiomyopathy [39].

### 2.3. Targeting Fatty Acid Oxidation in Cardiac Dysfunction

There are several strategies for improving fatty acid metabolism in cardiac conditions. First, it has been proposed to target modifiable behavioral risk factors. It has been shown that smoking cessation, improved diet, and physical activity improve cardiometabolic health and reduce cardiovascular risks [40] Second, dietary supplements such as coenzyme Q10, selenium, curcumin, omega-3 fatty acids, and vitamin D can improve cardiometabolic health by supporting the superstructural organization of enzymes, thus improving mitochondrial metabolism and reducing inflammation [41]. Short-chain fatty acid supplementation can potentially improve cardiac health [42]. Third, it is important to control underlying metabolic conditions such as diabetes. Fourth, management of genetic defects of fatty acid oxidation includes avoidance of prolonged fasting periods, dietary restriction of long-chain fats, supplementation with medium-chain triglycerides, and increase in carbohydrate intake [36]. Finally, the buildup of long-chain fatty acids increases hypercholesterolemia, and medication for lowering LDL and reducing atherosclerosis can be beneficial [43]. All these strategies do not target mitochondrial fatty acid oxidation directly. Recent studies provide several potential mitochondrial targets [44,45], however, additional mechanistic studies are required to identify the heart’s most critical defects and translate clinical findings into effective treatments.

## 3. Kidney

### 3.1. Essential Role of Fatty Acid Oxidation in Kidney Function

The normal glomerular filtration rate is 180–200 L daily. Measuring glomerular filtration rate is an important diagnostic procedure, and its decrease can indicate renal failure. About 660 mL of blood plasma flows through both kidneys per minute. Approximately 125 mL of filtrate is formed from this amount of plasma into the tubule lumen. The driving force that ensures filtration in the tubules is the transcapillary difference between hydrostatic and oncotic pressure [46]. However, the main role is played by the difference between the blood’s hydrostatic pressure and that of the Bowman-Schumlansky capsule. This is promoted by actively removing ions, glucose, and water from renal tubules, reducing the nephron’s hydrostatic and oncotic pressure.

**Figure 4:**
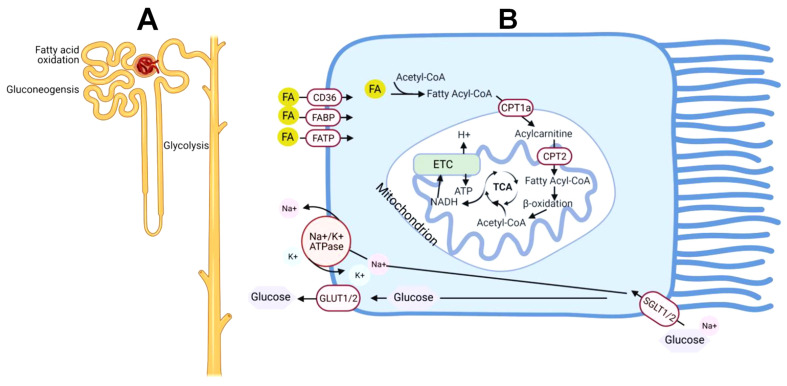
Schematic metabolism in the uninjured nephron and proximal tubule. (**A**) The proximal tubule segment has gluconeogenic capacity and preferentially uses fatty acid oxidation to generate ATP. By contrast, the distal tubules do not have gluconeogenic potential but are better equipped to generate ATP through glycolysis. (**B**) Schematic metabolism within the proximal tubule shows that glucose is taken up on the apical side by SGLT1/2 transporters and released on the basal side through GLUT1/2. Fatty acids (FA) cross the plasma membrane through CD36, fatty acid binding proteins (FABP), and fatty acid transport proteins (FATP). Carnitine shuttle involving carnitine palmitoyl-transferases CPT1a and CPT2 converts cytoplasmic fatty acids into acyl-CoA and transports them into the mitochondrial matrix. β-Oxidation of fatty acyl-CoA produces acetyl-CoA, which enters the TCA (tricarboxylic acid) cycle. Oxidation of acetyl-CoA by the TCA produces NADH, which enters the electron transport chain (ETC) to generate ATP. Created with BioRender.com.

The high number of mitochondria in the kidney cells and intensive metabolic processes of podocytes and renal tubular cells support the high respiratory activity of mitochondria. Mitochondria are unevenly distributed along the nephron. The highest density of mitochondria and the highest rates of fatty acid β-oxidation are found in the proximal tubule and thick ascending limb of the Henle loop (Figure 4) [47]. We will assume that in the kidney, mitochondria function similarly in podocytes or tubular epithelial cells. This is because the maximal rates of mitochondrial respiration are determined by the activities of enzymes located on the cytoplasmic membrane and cytosol.

The highest respiration and ATP demand rates occur in the first two segments of the proximal tubules. The respiratory activities of the kidney mitochondria were predominantly studied indirectly using isolated tubules of the nephrons [48,49] or by completely unrelated methods that give no information about the respiratory function of mitochondria, which is the central function of mitochondria [50]. However, kidney energy metabolism remains largely under-investigated at the mitochondrial level. To a large degree, this is because the β-oxidation of the long-chain fatty acids system in mitochondria has a very complex structure and requires the presence of supporting substrates that, in turn, depend on the animal’s metabolic phenotype, gender, and organ specificity [15,51]. Conversely, researchers study kidney mitochondrial metabolism using outdated paradigms based on carbohydrate catabolism, the “classical” structure of the respiratory chain, and incorrect methodology [49,50].

Nevertheless, data have accumulated over the years indicating that the kidney’s energy metabolism differs from other organs, which utilize long-chain fatty acids as the main energy source for ATP production. For example, unlike the heart, which extracts ^14^C-labelled palmitic, oleic, and linoleic acid at the same rate, palmitic acid is the only free fatty acid with a consistent net removal by the kidney [52]. Unlike heart and brain mitochondria with strong intrinsic inhibition of succinate dehydrogenase, kidney mitochondria oxidize succinate at high rates in all metabolic states [15,53]. However, the kidney mitochondria do not oxidize succinate during β-oxidation of the long-chain and middle-chain fatty acids. On the contrary, due to the reversal of the SDH reaction, succinate accumulates in the mitochondria, the tubular cells, and the blood. GPR91 protein, also called succinate receptor 1 (SUCNR1), faces the extracellular environment and responds to succinate with a half-maximum concentration of 28–56 µM. The highest succinate concentration reported for extracellular fluids was 200 µM [54]. GPR91 is expressed in many tissues, including blood cells, adipose tissue, the liver, the retina, and the kidney [55]. GPR91 and its succinate ligand are novel detectors of hypoxia and local ischemia, toxicity, and hyperglycemia. Local levels of succinate in the kidney also activate the renin-angiotensin system. It was suggested that GPR91 may play a vital role in developing hypertension and the harmful consequences of diabetes mellitus, metabolic disease, and liver damage [55].

The kidneys’ most energy-consuming function is associated with the reabsorption of sodium and glucose from the filtrate. Sodium-dependent glucose cotransporters (SGLT) located on the apical surface of the proximal tubule reclaim 180 g of glucose per day [56]. There are two forms of the SGLT. The low-affinity, high-capacity transporter (SGLT2) is located primarily in the S1 and S2 segments of the proximal tubule [57]. SGLT2 couples the transport of sodium and glucose in a 1:1 ratio and reabsorbs up to 90% of filtered glucose [58]. SGLT1 is a high-affinity, low-capacity transporter located in the S3 segment of the proximal tubule and transports sodium in a 2:1 ratio with glucose [59]. Normally, the kidney metabolizes very little glucose, allowing glucose to move down a concentration gradient back into circulation. GLUT2 is the transporter found in S1 and S2 proximal tubule segments that match the high-capacity, low-affinity glucose flux initiated by SGLT2 on the apical surface. Similarly, GLUT1 provides an exit pathway for glucose that enters the S3 segment ATPase, which also consumes a lot of ATP in a short time. Thus, the proximal tubule mitochondria rely primarily on the β-oxidation of long-chain fatty acids for energy. Normally, kidneys operate at 60% of the maximal capacity to produce energy [48]. However, under the conditions of chronic hyperglycemia and high sodium consumption, the kidneys may operate at the maximum of their capacity to produce ATP, and this situation may result in the development of local hypoxia that leads to acute kidney injury (AKI) and then chronic kidney disease (CKD) [60].

### 3.2. Impaired Fatty Acid Oxidation in Kidney Dysfunction

Unlike other organs, which respond to increased functional load by increasing blood flow, for the kidneys, an increase in blood flow means an increase in the need for oxygen. The oxygen content (P_O2_) in the kidney tissue is relatively independent of renal blood flow within ±30%, which cannot occur in other organs such as the brain or heart [61]. This is because, in the Bowman–Schumlansky capsule, oxygen is exchanged between arteries and veins, which increases with rising blood flow. Thus, local hypoxia occurs in chronic hyperglycemia and high dietary sodium intake, leading to local proximal tubular damage. It has recently been shown that β-oxidation of fatty acids is a highly complex functional superstructure [51], vulnerable to hypoxia. Local hypoxia leads to a local increase in perhydroxyl radical (a protonated form of superoxide) and activation of the isoprostane lipid peroxidation, which causes damage to mitochondrial hyperstructures. This hypothesis explains why most kidney pathologies start with lipid metabolism disorder [62,63].

### 3.3. Targeting Fatty Acid Oxidation in Kidney Dysfunction

Given our limited knowledge of the kidney’s lipid metabolism, at the present time, it is difficult to discuss how specifically impaired fatty acid oxidation may cause kidney dysfunction. We suggest that the accumulation of fatty acids occurs due to oxidative damage to the respirasome function. Supplementation with antioxidants can potentially reduce kidney damage [64,65,66], however, most of clinical studies do not support the therapeutic effects of “common” antioxidants [67], which is likely due to the impairment of intrinsic antioxidants such as mitochondrial superoxide dismutase [68]. One of the best strategies for avoiding kidney dysfunctions is to prevent oxidative stress by ameliorating blood sugar and controlling sodium consumption.

## 4. Skeletal Muscle

### Essential Role of Fatty Acid Oxidation in Skeletal Muscle Function

Striated muscle exists in two major muscle types: skeletal and cardiac. While the cardiac muscle functions largely as a syncytium comprising non-fatiguing muscle cells, skeletal muscle represents a set of well-innervated muscle cells that exhibit fatigue under conditions of high energy requirements. A cellular molecular structure and molecular crosstalk allow us to appreciate the complexity of striated muscle’s composition, structure, and function [69]. Metabolic events, particularly those responsible for energy supply, are full of contradictions and ambiguities. Over the decades, reviews in skeletal muscle research have focused extensively on specific aspects of muscle structure or function under various physical activity levels, which might vary from almost full inactivity to maximum activity. Correspondingly, this requires substrate adaptation for each level of physical activity. This review emphasizes that β-oxidation of long-chain fatty acids lies at the core of all muscle and body metabolic events. Under conditions of low physical activity, oxidative phosphorylation relies on the oxidation of pyruvate produced by aerobic glycolysis and anaplerotically from alanine and α-ketoglutarate by alanine transaminase (ALT) [70]. The β-oxidation of fatty acids provides energy and carbons, and pyruvate also serves as a supporting substrate for this β-oxidation [71]. Endurance training also elicits mitochondrial adaptations that enhance fatty acid oxidation capacity [72]. Under these conditions, fatty acids completely replace pyruvate as a main substrate for ATP production. Therefore, pyruvate reduces to lactate and accumulates in the muscles and blood [73].

We have already stressed that the oxidative metabolism of long-chain and middle-chain fatty acids remains poorly investigated. During the last decade, several great discoveries have been made, which suggest that activation of fatty acid oxidative metabolism requires the simultaneous presence of supporting substrates and that the enzymes involved in the process are organized into very complex superstructures [15,51]. Brand and colleagues discovered that β-oxidation of palmitoyl-carnitine by the skeletal muscle mitochondria generates the highest level of reduction of the coenzyme Q in mitochondria, reverses the flow of electrons from ubiquinol at the level of succinate dehydrogenase (complex II), and reduces fumarate to succinate [74,75,76]. Wang et al. provided evidence for physical association of mitochondrial fatty acid oxidation and oxidative phosphorylation complexes [77].

No longer conceived of as a dead-end metabolite, a fatigue agent, or metabolic poison, lactate is seen in contemporary physiology as a major metabolic intermediate that has wide-ranging impacts on energy substrate utilization, cell signaling, and adaptation; it is at the fulcrum of metabolic integration [78]. We suggest that under stressful conditions, when the body performs heavy physical work actively oxidizing long-chain fatty acids, the lactate released into the blood by hard-working skeletal muscles serves as an emergency substrate for other organs, such as the heart and brain.

## 5. Endothelial Cells and Vascular Function

### 5.1. Fatty Acid Oxidation in Endothelial Cells

The current dogma is that endothelial cells rely on glycolysis instead of mitochondrial respiration, and mitochondria are not critical for endothelial metabolism [79]. Indeed, endothelial and smooth muscle cells in the vasculature are mainly present in a quiescent phenotype and do not have a high energy demand like the heart, kidney, or skeletal muscle described above. This dogma, however, is based mainly on in vitro studies in cell culture, which is prone to artifacts, particularly if streptomycin is present in the culture media [70]. Recent studies show that “all mitochondria are created equal”, indicating that the oxidative phosphorylation capacity per mitochondrial content in vascular cells is similar to that in cardiac and skeletal muscle mitochondria [80]. Therefore, reduced oxygen consumption by vascular cells can be explained by diminished mitochondrial content compared to cardiomyocytes and skeletal muscle. Furthermore, fatty acid oxidation and mitochondrial ATP production are important in endothelial homeostasis and endothelial-dependent relaxation [81,82]. However, the role of mitochondria in endothelial function has not been well-studied. Recent studies showed equal utilization of glycolysis and mitochondrial respiration by vascular cells [83], which are normally coupled [84], meaning that glycolysis product pyruvate is utilized by mitochondria. Mitochondrial dysfunction leads to uncoupled glycolysis, i.e., glycolysis is disproportionately increased compared with mitochondrial respiration, which can be observed in cell cultures where metabolism is skewed towards glycolysis. Vascular cells use both mitochondrial oxidative phosphorylation and glycolysis [83]. However, inhibition of mitochondrial metabolism leads to a maladaptive switch to glycolysis, adversely affecting cell function and phenotype [81]. The pathophysiological role of the glycolytic switch in vascular smooth muscle cells and adventitial fibroblasts [85] promotes aortic aneurysms and vascular fibrosis [86]. In endothelial cells, the glycolytic switch promotes phagocytosis of gap junctions, increasing endothelial barrier permeability and inducing the endothelial–mesenchymal transition [81]. Dr. Finkel’s group showed that genetic ablation of the acyl-carnitine transporter is sufficient for these metabolic and phenotypic alterations, demonstrating a key role of mitochondrial fatty acid oxidation in endothelial cell function [81]. Fatty acid oxidation has been implicated in endothelial barrier transport. Dr. Arany and colleagues showed that mitochondrial but not glycolytic ATP regulates endothelial fatty acid uptake and transport [87]. which is critical for perivascular cells and end-organ tissue. These data support the critical role of fatty acid oxidation in endothelial-barrier function, transendothelial transport, endothelial phenotype maintenance, and vasorelaxation. Meanwhile, endothelial fatty acid metabolism is not well understood. The specific functions of long-chain, medium-chain, and short-chain fatty acids in endothelial cells are unclear. We also do not know the interplay between fatty acid oxidation pathways and bioactive metabolites of polyunsaturated fatty acids, including arachidonic acid, eicosapentaenoic acid, and docosahexaenoic acid [88]. Additional studies of these pathways can provide new insights into molecular mechanisms of endothelial homeostasis and function.

### 5.2. Impaired Fatty Acid Oxidation in Endothelial Dysfunction

Alterations of several fatty acid metabolism pathways contribute to endothelial and vascular dysfunction. First, direct exposure of endothelium to high levels of fatty acids in circulation is detrimental and promotes endothelial dysfunction and inflammation [89,90]. Second, CD36-dependent endothelial fatty acid transport alterations have been implicated in metabolic abnormalities and cardiovascular disease [91]. Third, the role of carnitine palmitoyl transferase alterations in endothelial dysfunction has been reported [81,92]. Finally, it has been suggested that mitochondrial dysfunction can drive metabolic reprogramming of vascular endothelial cells in pulmonary hypertension and other cardiovascular conditions; however, these pathways are incompletely characterized [93]. Additional studies are required to resolve conflicting reports of reduced or increased endothelial glycolysis in endothelial dysfunction [94,95] and define the specific alterations of mitochondrial fatty acid in specific vascular conditions, which may have opposite directions depending on the stage of the disease [93].

### 5.3. Targeting Fatty Acid Oxidation in Endothelial Dysfunction

Several targets to improve vascular and endothelial metabolism have been suggested. First, targeting glycolysis can potentially increase mitochondrial metabolism and cell function, for example, by using the PDK inhibitor dichloroacetate [96] and PFKFB3 blocker 3-(3-pyridinyl)-1-(4-pyridinyl)-2-propen-1-one [97], however, this approach does not provide tissue-specific inhibition of glycolysis, which may cause off-target effects. Second, metabolic pathways can be targeted by metformin [98] and an inducer of fatty acid oxidation, PPARα agonist fenofibrate [99]. Meanwhile, it is still debated whether inhibiting fatty acid oxidation in endothelial cells causes cell senescence or can potentially promote endothelial cell proliferation [92,99]. Specific defects in vascular mitochondrial fatty acid oxidation are still elusive, and additional studies can potentially uncover novel therapeutic approaches to correct these metabolic defects in endothelial and vascular dysfunction.

## 6. Epithelial Cells

### 6.1. Fatty Acid Oxidation in Epithelial Cell Function

Epithelial cells provide a physical barrier in the skin, lungs, gastrointestinal tract, and genitourinary system. Mitochondria play a key role in epithelial cell homeostasis [100]. Epithelial cells provide tight epithelial-barrier, sensory, transportation, absorption, and secretion functions [101]. Despite the great variety in structure and function of these cells, there is growing evidence for the important role of fatty acid oxidation in epithelial metabolism. For example, human airway epithelium vividly absorbs long-chain fatty acids and metabolizes them within a few hours [102]. Intestinal epithelial cells change their fatty acid oxidation depending on food intake and gut microbiota [103]. It has been shown that maintaining a tight barrier in intestinal epithelial cells depends on mitochondrial oxidative phosphorylation [104]. Interestingly, medium-chain fatty acids were more effective in maintaining epithelial ATP production than long-chain fatty acids (C16:0 and C18:0) [105]. Epithelial cells are constantly regenerating, which requires metabolic rewiring towards fatty acid oxidation, and modulation of fatty acid oxidation influences epithelial cell differentiation [106]. These cells mainly rely on mitochondrial respiration but can also use glycolysis under basal conditions [104]. In hypoxia, epithelial cells undergo a metabolic switch toward glycolysis. However, enhanced glycolysis is linked to epithelial-mesenchymal transition, inflammation, and cancer [107,108].

### 6.2. Impaired Fatty Acid Oxidation in Epithelial Dysfunction

The high-fat diet causes a metabolic shift toward fatty acid β-oxidation in small intestine epithelial cells. It impairs colonocyte mitochondrial function, potentially due to deleterious metabolites produced by gut microbiota [109]. Genetic alterations of mitochondrial function can drive the epithelial-to-mesenchymal transition and contribute to epithelial dysfunction. Reduced expression of carnitine palmitoyltransferase 1, SLC22A5 encoded carnitine transporter OCTN2, immunity-related GTPase-linked mitochondrial fission, and CypD-mediated mitochondrial permeability transition pore opening impair mitochondrial fatty acid oxidation, promote inflammation, and contribute to epithelial dysfunction [110].

### 6.3. Targeting Fatty Acid Oxidation in Epithelial Dysfunction

Proinflammatory cytokines, such as TNF-α, downregulate the expression of PGC-1α, carnitine palmitoyltransferase 1A (CPT1A), long-chain acyl-CoA dehydrogenase (LCAD), and medium-chain acyl-CoA dehydrogenase (MCAD) in alveolar epithelial cells leading to impairment of fatty acid oxidation, reduced regeneration capacity, and cell apoptosis [111]. Interestingly, the PPAR-α agonist fenofibrate improves the expression of PGC-1α, CPT1A, LCAD, and MCAD and attenuates LPS-induced acute lung injury [111]. Lipid accumulation is implicated in diabetic nephropathy, reflecting an imbalance between fatty acid utilization and supply. Lipotoxicity caused by fatty acid accumulation and tubule-interstitial fibrosis linked to epithelial-to-mesenchymal transition are the hallmarks of diabetic nephropathy. Interestingly, silencing the acetyl-CoA carboxylase 2, a key enzyme for fatty acid biosynthesis, increased the β-oxidation rate, reduced lipotoxicity, and diminished epithelial-to-mesenchymal transition [112].

## 7. Discussion and Final Remarks

In this paper, we have discussed that mitochondrial fatty acid oxidation is essential not only in energy-demanding organs such as the heart, skeletal muscle, and kidney but also for functions in metabolically “inactive” organs, such as endothelial and epithelial cells. It is important that fatty acid oxidation plays a critical role in the function of our body regardless of our diet, and even a “fat-free” vegetarian diet will be converted into fatty acids as a primary fuel source and essential cell membrane building blocks. Meanwhile, increased fatty acid dietary consumption, low physical activity, and metabolic disorders lead to detrimental fatty acid accumulation, promoting cell dysfunction, inflammation, and organ damage. Analysis of the literature suggests that impaired fatty acid oxidation contributes to many pathological conditions such as arterial hypertension, pulmonary hypertension, heart disease, atherosclerosis, inflammation, kidney dysfunction, skeletal muscle pain, and weakness. Despite several decades of studies, there is a significant gap of knowledge in understanding the specific molecular mechanisms leading to impaired fatty acid mitochondrial metabolism. We do not know what causes the decline in fatty acid oxidation in many pathological conditions and how we can rescue and improve the function of mitochondria in these conditions. Of particular importance are accumulating data that β-oxidation of fatty acids requires the presence of amino acids and some mitochondrial intermediates, which strongly depends on gender and metabolic phenotype [113,114]. In addition, recently, it was suggested that as a metabolic pathway, β-oxidation of fatty acids is organized into a very large supercomplex, which structurally and functionally works as one and critically depends on mitochondrial cardiolipin and phosphatidylethanolamine [15]. This makes fatty acids’ oxidative metabolism highly sensitive to oxidative stress. Further studies are urgently needed to define these pathophysiological mechanisms to improve the treatment of emerging crises with metabolic conditions.

In this work, we did not discuss the important role of fatty acid metabolism in the pancreatic islet β-cells function [115], the complex role of lipid metabolism in the regulation of inflammation and immune cell function [116], potential targeting of mitochondrial fatty acid oxidation in therapeutic interventions in cancer [117], and the role of fatty acid oxidation in the brain. These are new and promising emerging fields; however, specific molecular mechanisms under these physiological and pathophysiological conditions remain poorly understood, and many questions are still waiting to be answered. In this respect, our work does not comprehensively answer all the questions on the physiological and pathological role of fatty acids. Rather, we raise awareness of the urgent need for a paradigm shift, existing knowledge gaps, and the significance of future studies.
